# A Case Series Tries to Answer Whether Exercise Positively Influences Immunosenescence

**DOI:** 10.7759/cureus.51072

**Published:** 2023-12-25

**Authors:** Binoy M K V, Jagatheesan Alagesan

**Affiliations:** 1 Physiotherapy, Saveetha College of Physiotherapy, Saveetha Institute of Medical and Technical Sciences, Chennai, IND

**Keywords:** ageing and frailty, case-series, cd8 cells, cd4 t-cells, long term care, muscular balance, elderly individuals, elderly population, immunosenescence, exercise immunology

## Abstract

The immune system, the defense mechanism of the body, mainly consists of lymphocytes. The three primary subtypes of lymphocytes are natural killer cells (NK cells), bone marrow-derived lymphocytes (B-lymphocytes), and thymus-derived lymphocytes (T-lymphocytes). T-lymphocytes are mostly composed of CD (cluster of differentiation) cells, such as CD4+ and CD8+ subsets of CD cells. Immunosenescence is the term for the steady decline of the immune system with aging. There are alterations in the composition of various types of lymphocytes, especially in CD4 and CD8 T cells. The elderly are more vulnerable to infections due to immunosenescence, which raises morbidity and mortality rates. Physical exercises are believed to have the potential to alter immunosenescence and produce positive changes in immune cell composition.

The extent of exercise-induced immune function changes was different in research studies owing to the differences in protocols, methodologies, testing procedures, ages, and gender compositions. The impact of an eight-week balance-based exercise intervention on immune biomarkers is investigated in this case series. In this case series, two elderly women residing in an institutional setting were exposed to an eight-week-long balanced-based supervised exercise intervention. The immunological biomarkers CD45, CD3, CD4, and CD8 were the outcome variables that were assessed. Data on the outcome variables was gathered both before and after the intervention. Using flow cytometry and single-platform technology, immune biomarkers were analyzed. Following the intervention, there was a rise in the CD45, CD3, CD4, and CD8 cell counts relative to the baseline data. The biomarkers showed only slight and statistically non-significant improvements.

## Introduction

The immune system is the defense mechanism of the body. The primary functions of the immune system are identification with subsequent destruction of foreign antigens, immunological memory formation, and tolerance development to antigens inherent to our body [1]. This defensive system of the body consists of lymphocytes. The main types of lymphocytes are thymus-derived lymphocytes (T-lymphocytes), bone marrow-derived lymphocytes (B-lymphocytes), and natural killer cells (NK cells). T-lymphocytes lead cellular immunity, and B-lymphocytes mediate humoral immunity. The cluster of differentiation (CD) cells, including CD4+ and CD8+, form the majority of T-lymphocytes [1]. CD 45 plays a significant role in T-cell receptor activation by acting as a signaling gatekeeper [2]. CD4+ T-cells play a key role in the activation of cells of the innate immune system, cytotoxic T-cells, B-lymphocytes, and suppression of immune reaction [1]. CD8+ T-cells play a significant role against intracellular pathogens, including viruses and bacteria [3]. Naïve cells denote cells that are active young cells that are not exposed to antigens, and effector memory cells are those that have been exposed to antigens before.

The mean normal values and percentage of CD4+ lymphocytes in healthy Indian adults were 865 cells/μl (40.2%), and for CD8+ lymphocytes, were 552 cells/μl (31.3%) and 1.7 for the CD4:CD8 ratio [4]. The normal CD4 subset cell value found in Chinese adults was 844 ± 247 cells/μl, and the CD8 lymphocyte subset value was 539 ± 134 cells/μl. The CD4/CD8 ratio found was 1.49 with a standard deviation of 0.57 [5].

There is a progressive deterioration of the immune system related to aging known as immunosenescence. Immunosenescence makes the elderly susceptible to infections, leading to an increase in morbidity and mortality. Immunosenescence is caused by multiple factors, like genetic and environmental factors, unhealthy lifestyles, and long-standing psychological stress. It was reported that there is a quantitative and qualitative decline in naïve and effector memory CD4 T cells, CD8+, and αβ T-cell subsets as age progresses in older people. Senescent CD4+ T cells increase at a rate of 10% per decade, and senescent CD8+ T-cells increase at a rate of 10.2% per decade. Also, there is a progressive reduction of naïve CD4+ T-cells at the rate of 10% per decade and naïve CD8+ T-cells at the rate of 9.9% per decade [6]. There is an inversion of the CD4/CD8 ratio (CD4:CD8 ratio <1), shortening of the telomere, and oligoclonal expansion of virus-specific T-cells [7,8].

Physical exercise is considered a potential strategy for addressing immunosenescence. Moderate levels of exercise have a beneficial effect on addressing immunosenescence. Exercise interventions improve T-cell activity among the elderly. Moderate levels of regular exercise improve immunity by reducing inflammation and alterations in the composition of young and older immune cells, maintaining thymic mass, enhancing immunosurveillance, and reducing psychological stress [9]. Aerobic exercise has shown a greater effect on improving the immune system. The degree to which exercise altered immune function varied between research studies, which can be attributed to variations in testing procedures, age and gender distribution, protocols, and methodologies. So, we conducted an eight-week supervised balance-based aerobic exercise program and evaluated the immunity biomarker changes in two cases in this case series.

## Case presentation

Case series

Two elderly women residing in an institutional setting were included in this case series. Eligibility criteria were age more than 60 years old, independence in ambulation, and ability to follow oral instructions. Relevant details of the cases are given below.

Case 1

The first case was an elderly woman, 68 years old, with an average build who had spent the previous four years in the facility. She took oral hypoglycemic medications on a regular basis due to her diabetes. She wore corrective glasses since she had a slight visual impairment. She became unsteady when turning, and she walked extremely cautiously outside. She could barely ascend because of weakness around her knees. She sits in the commode and stands using the grab grips for support. With a score of 1, she demonstrated a low level of physical performance on the Short Physical Performance Battery (SPPB). Her Fall Efficacy Scale-International (FES-I) score of 36 indicated a high level of anxiety about falling, and her Four Square Step Test (FSST) duration of 33.81 seconds indicated a high fall risk.

Case 2

The second case included an elderly woman of sixty-five years of slender figure who had spent the previous two years at the facility. Her vision impairment was mild, and she regularly wore corrective glasses. She was prescribed medication for hypertension, diabetes, and high cholesterol on a regular schedule. With a score of 7, she demonstrated a modest level of physical performance on the Short Physical Performance Battery (SPPB). Her fall risk was modest as evidenced by her Four Square Step Test (FSST) time of 10.91 seconds and her Fall Efficacy Scale-International (FES-I) score of 24, which showed moderate concern about falling.

**Figure 1:**
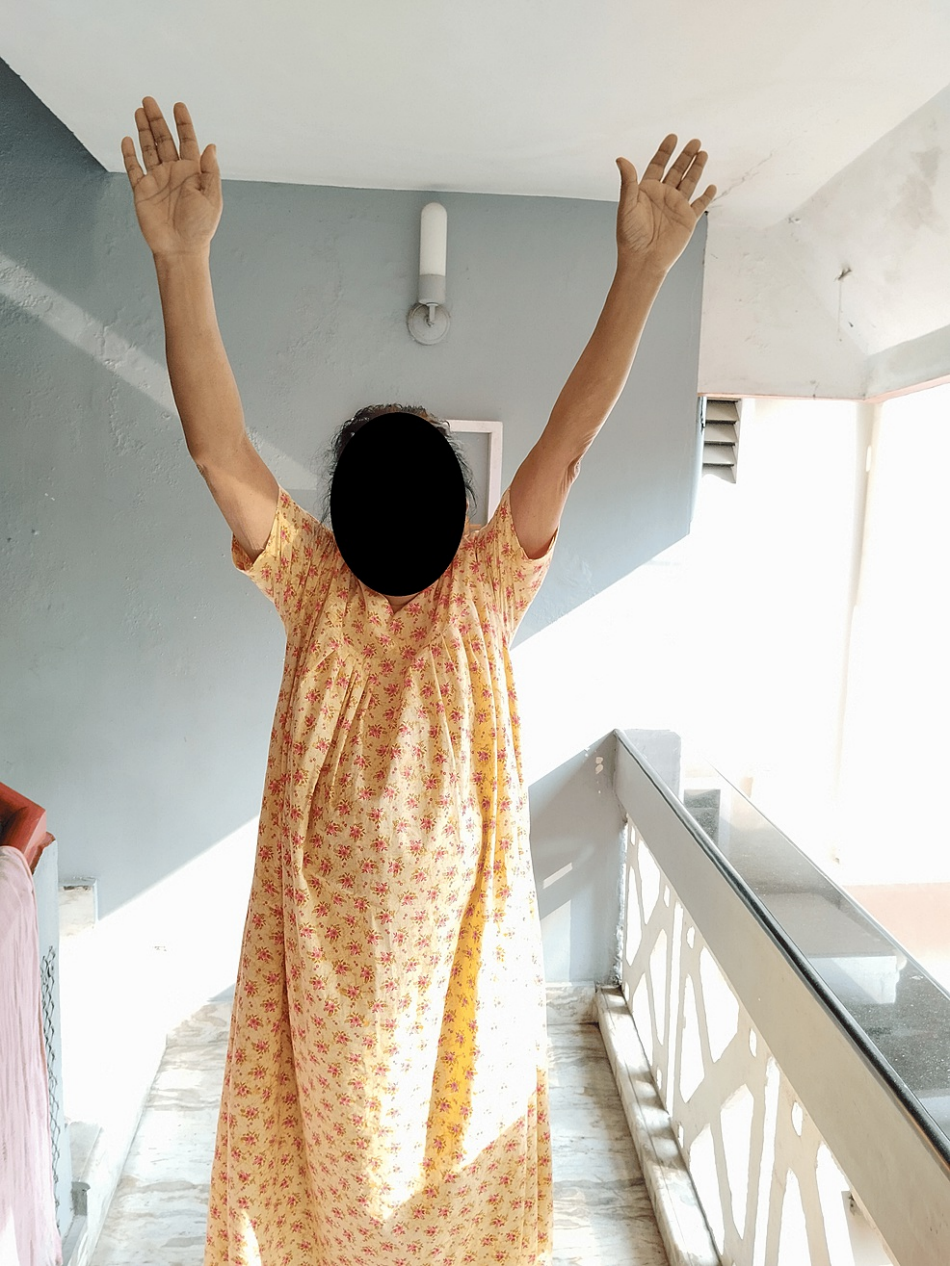
Subject undergoing balance-based aerobic intervention program

Informed consent was obtained from the subjects prior to the commencement of the research work. Institutional ethical clearance was obtained (IEC/2021/5/4). Demographic details were collected from the subjects. The outcome measures of immunity were immunity biomarkers such as CD45, CD3, CD4, and CD8. The immunity biomarkers were collected at baseline and after an eight-week exercise intervention. The supervised, balance-based aerobic exercise intervention of 30-minute duration was provided three times per week for eight weeks (Figure 1). The exercise program consisted of a five-minute warm-up exercise, followed by forward walking, backward walking, walking sideward, heel walking, toe walking, and single-leg balancing with an adequate rest period in between. The exercise program ceased with a five-minute cool-down exercise.

Analysis of immunity biomarkers was conducted using flow cytometry single-platform technology. The procedure was conducted with a BD FACS Canto II automated 6-color flow cytometer (BD, Franklin Lakes, New Jersey, United States) using Canto clinical software (BD, Franklin Lakes, New Jersey, United States). Specimens were sequentially gated in the order of CD45 leukocyte common antigen, CD3 T-cells, CD4 helper T-cells, and CD8 suppressor T-cells. The absolute count and individual percentages were determined, along with the CD4:CD8 ratio as shown in Table 1.

**Table 1:** Immunity biomarker analysis results

Cases	Immunity Biomarkers	Baseline	Post Intervention	Biological Reference Interval
Case-1	CD45 Absolute	1705 cells/mm3	1786 cells/mm3	1115-4009 cells/mm3
Case-2	CD45 Absolute	1113 cells/mm3	1142 cells/mm3	1115-4009 cells/mm3
Case-1	CD3 T Cells %	59.49%	59.53%	55-81%
Case-2	CD3 T Cells %	69.29%	69.34%	55-81%
Case-1	CD3T Cells Absolute	1014 cells/mm3	1063 cells/mm3	457-3926 cells/mm3
Case-2	CD3T Cells Absolute	771 cells/mm3	779 cells/mm3	457-3926 cells/mm3
Case-1	CD4 Helper T Cells %	37.91%	37.83%	27-51%
Case-2	CD4 Helper T Cells %	39.16%	39.08%	27-51%
Case-1	CD4 Helper T Cells Absolute	647 cells/mm3	676 cells/mm3	448-1611 cells/mm3
Case-2	CD4 Helper T Cells Absolute	436 cells/mm3	451 cells/mm3	448-1611 cells/mm3
Case-1	CD8 Suppressor T Cells %	21.33%	21.01%	20.06-42.52%
Case-2	CD8 Suppressor T Cells %	31.29%	31.23%	20.06-42.52%
Case-1	CD8 Suppressor T Cells Absolute	364 cells/mm3	375 cells/mm3	218-1396 cells/mm3
Case-2	CD8 Suppressor T Cells Absolute	348 cells/mm3	356 cells/mm3	218-1396 cells/mm3
Case-1	CD4:CD8 Ratio	1.78	1.8	0.39-3.02
Case-2	CD4:CD8 Ratio	1.25	1.31	0.39-3.02

## Discussion

Exercise has shown a significant effect on immunity, as demonstrated by the positive effects on immunosenescence, cancer, viral infections, and inflammatory diseases [10]. In the current case series, there was some positive increase in CD45 cells (55 + 39.59 SD) in both cases, suggesting an activation of the immune process. It was reported that a single session of cardiorespiratory or resistance exercise enhanced the count of CD45+ cells [11].

There was a minimal increase in the CD3 absolute count (30 + 31.11 SD) and percentage in both cases. However, the changes found were not statistically significant. A slight decrease in the percentage of CD4 helper T-cells and CD8 suppressor T-cells was found following the intervention. The minimal increase in the number of CD4 helper cells (22 + 9.89 SD) and CD8 suppressor T-cells (9.5 + 2.12 SD) reported after the intervention was insignificant. Similar results were reported in a 12-week study of aerobic and resistance exercise interventions that showed no significant change in CD3+, CD4+, and CD8+ T-cells [12]. In both cases, there was an increase in the CD4:CD8 ratio, suggesting a proportional prevalence of CD4 helper T-cells versus CD8 suppressor T-cells after the intervention. Other studies have reported that regular moderate-intensity exercise lasting less than 60 minutes leads to improvements in immune function [13]. There is an attenuation of age-related deterioration of T-cells, B-cells, and NK cells with regular moderate-intensity exercise [14]. A randomized controlled trial among cognitively impaired seniors reported immunomodulation of CD4+ and CD8+ following a year of moderate-intensity exercise [15].

The immune, endocrine, and central nervous systems are closely interlinked. The main mechanism for positive immune modulation is a catecholamine-induced β-adrenergic receptor (β-AR) signaling in leukocytes [16]. A theory has been proposed to explain the processes involved in immunity changes in response to exercise. The three-step process explains how exercise influences the preventive and restorative mechanisms of T-cell immunosenescence. The first stage involves the mobilization of T-cells at the late stage of differentiation (senescent T-cells) into peripheral blood during exercise. In the second process, there is extravasation of T-cells from peripheral and inflamed cells, usually one to two hours after exercising. Then these cells are exposed to a variety of pro-apoptotic stimuli, leading to the apoptosis or death of these cells. During the third and final process, the naïve T-cell repertoire then occupies the immune space created by the destruction of senescent T-cells and boosts the immunity system [6,17-19].

The limitation is that meaningful statistical inferences could not be derived from the case series. An experimental study with an adequate sample size is recommended.

## Conclusions

A moderate level of exercise was reported as producing positive changes in immunosenescence. The current case series among institutionalized elderly patients produced a marginal increase in CD45, CD3, CD4, and CD8 T-cells after exercise intervention for eight weeks. There was a positive modification in the CD4:CD8 ratio. However, the change was minimal and statistically insignificant. The results suggest that a positive influence on immune biomarkers is possible with a balance-based moderate-intensity aerobic exercise program of an eight-week duration. Considering the limitations of the case series, randomized controlled trials with a large sample size are recommended among the elderly to substantiate the positive effect of physical exercise on immunosenescence.

## References

[1] Luckheeram RV, Zhou R, Verma AD, Xia B (2012). CD4⁺T cells: differentiation and functions. Clin Dev Immunol.

[2] Rheinländer A, Schraven B, Bommhardt U (2018). CD45 in human physiology and clinical medicine. Immunol Lett.

[3] Sigal LJ (2016). Activation of CD8 T lymphocytes during viral Infections. Encyclopedia of Immunobiology.

[4] Uppal SS, Verma S, Dhot PS (2003). Normal values of CD4 and CD8 lymphocyte subsets in healthy indian adults and the effects of sex, age, ethnicity, and smoking. Cytometry B Clin Cytom.

[5] Jiang W, Kang L, Lu HZ (2004). Normal values for CD4 and CD8 lymphocyte subsets in healthy Chinese adults from Shanghai. Clin Diagn Lab Immunol.

[6] Minuzzi LG, Rama L, Chupel MU (2018). Effects of lifelong training on senescence and mobilization of T lymphocytes in response to acute exercise. Exerc Immunol Rev.

[7] Pera A, Campos C, López N, Hassouneh F, Alonso C, Tarazona R, Solana R (2015). Immunosenescence: implications for response to infection and vaccination in older people. Maturitas.

[8] Silva LC, de Araújo AL, Fernandes JR (2016). Moderate and intense exercise lifestyles attenuate the effects of aging on telomere length and the survival and composition of T cell subpopulations. Age (Dordr).

[9] Simpson RJ, Kunz H, Agha N, Graff R (2015). Exercise and the regulation of immune functions. Prog Mol Biol Transl Sci.

[10] Simpson RJ, Boßlau TK, Weyh C, Niemiro GM, Batatinha H, Smith KA, Krüger K (2021). Exercise and adrenergic regulation of immunity. Brain Behav Immun.

[11] Graff RM, Jennings K, LaVoy EC, Warren VE, Macdonald BW, Park Y, Markofski MM (2022). T-cell counts in response to acute cardiorespiratory or resistance exercise in physically active or physically inactive older adults: a randomized crossover study. J Appl Physiol (1985).

[12] Shimizu K, Suzuki N, Imai T (2011). Monocyte and T-cell responses to exercise training in elderly subjects. J Strength Cond Res.

[13] Nieman DC, Wentz LM (2019). The compelling link between physical activity and the body's defense system. J Sport Health Sci.

[14] Burtscher J, Pasha Q, Chanana N, Millet GP, Burtscher M, Strasser B (2023). Immune consequences of exercise in hypoxia: a narrative review. J Sport Health Sci.

[15] Poinsatte K, Smith EE, Torres VO (2019). T and B cell subsets differentially correlate with amyloid deposition and neurocognitive function in patients with amnestic mild cognitive impairment after one year of physical activity. Exerc Immunol Rev.

[16] Hong S, Dimitrov S, Pruitt C, Shaikh F, Beg N (2014). Benefit of physical fitness against inflammation in obesity: role of beta adrenergic receptors. Brain Behav Immun.

[17] Simpson RJ (2011). Aging, persistent viral infections, and immunosenescence: can exercise "make space"?. Exerc Sport Sci Rev.

[18] Simpson RJ, Guy K (2010). Coupling aging immunity with a sedentary lifestyle: has the damage already been done?--a mini-review. Gerontology.

[19] Simpson RJ, Cosgrove C, Chee MM (2010). Senescent phenotypes and telomere lengths of peripheral blood T-cells mobilized by acute exercise in humans. Exerc Immunol Rev.

